# Early identification and preventive care for elevated cardiovascular disease risk within a remote Australian Aboriginal primary health care service

**DOI:** 10.1186/1472-6963-11-24

**Published:** 2011-01-31

**Authors:** Christopher P Burgess, Ross S Bailie, Christine M Connors, Richard D Chenhall, Robyn A McDermott, Kerin O'Dea, Charlie Gunabarra, Hellen L Matthews, Adrian J Esterman

**Affiliations:** 1 Menzies School of Health Research, Institute of Advanced Studies, Charles Darwin University, Darwin, Australia; 2 Northern Territory Clinical School, Flinders University, Darwin, Australia; 3 Department of Health and Families, Northern Territory Government, Darwin, Australia; 4 Centre for Health and Society, Melbourne School of Population Health, University of Melbourne, Melbourne, Australia; 5 Sansom Institute for Health Research, University of South Australia, Adelaide, Australia

## Abstract

**Background:**

Cardiovascular disease (CVD) is the single greatest contributor to the gap in life expectancy between Indigenous and non-Indigenous Australians. Our objective is to determine if holistic CVD risk assessment, introduced as part of the new Aboriginal and Torres Strait Islander Adult Health Check (AHC), results in better identification of elevated CVD risk, improved delivery of preventive care for CVD and improvements in the CVD risk profile for Aboriginal adults in a remote community.

**Methods:**

Interrupted time series study over six years in a remote primary health care (PHC) service involving Aboriginal adults identified with elevated CVD risk (N = 64). Several process and outcome measures were audited at 6 monthly intervals for three years prior to the AHC (the intervention) and three years following: (i) the proportion of guideline scheduled CVD preventive care services delivered, (ii) mean CVD medications prescribed and dispensed, (iii) mean PHC consultations, (iv) changes in participants' CVD risk factors and estimated absolute CVD risk and (v) mean number of CVD events and iatrogenic events.

**Results:**

Twenty-five percent of AHC participants were identified as having elevated CVD risk. Of these, 84% had not been previously identified during routine care. Following the intervention, there were significant improvements in the recorded delivery of preventive care services for CVD (30% to 53%), and prescription of CVD related medications (28% to 89%) (*P *< 0.001). Amongst participants there was a 20% relative reduction in estimated absolute CVD risk (*P *= 0.004) following the intervention. However, there were no significant changes in the mean number of PHC consultations or mean number of CVD events or iatrogenic events.

**Conclusions:**

Holistic CVD risk assessment during an AHC can lead to better and earlier identification of elevated CVD risk, improvement in the recorded delivery of preventive care services for CVD, intensification of treatment for CVD, and improvements in participants' CVD risk profile. Further research is required on strategies to reorient and restructure PHC services to the care of chronic illness for Aboriginal peoples in remote areas for there to be substantial progress in decreasing excess CVD related mortality.

## Background

Cardiovascular disease (CVD) is the leading cause of excess mortality and the greatest single contributor to the burden of disease for Aboriginal and Torres Strait Islander Australians [1,2]. Between 25-54 years of age, CVD deaths for Indigenous Australians occur at 9 to 12 times the rate for non-Indigenous Australians [2].

The Aboriginal and Torres Strait Islander Adult Health Check (AHC) was launched by Australia's federal Government in 2004. The objective of the AHC is "to encourage early detection, diagnosis and intervention for common and treatable conditions that cause considerable morbidity and early mortality" [3]. The AHC comprises a pre-determined suite of preventive clinical services including the holistic assessment of CVD risk [3]. Upon completion of an AHC, General Practitioners (GPs) are rewarded with a fee for service. Applicable annually between 15 to 54 years of age, the AHC is a key component of the strategy to 'close-the-gap' in life expectancy between Australia's Indigenous peoples and the general population [4,5]. CVD risk assessment within the AHC has the potential to improve the early detection and preventive care for CVD, particularly in remote areas, where CVD contributes disproportionately to the disease burden [1].

Our objective was to determine if CVD risk assessment during an AHC results in (i) better identification of elevated CVD risk, (ii) improved delivery of preventive services for CVD and (iii) improvement in the CVD risk profile for Aboriginal adults in a remote Australian Aboriginal primary health care service.

## Methods

### Setting and Participants

The study setting was a large Arnhem Land community in Australia's Northern Territory (NT). Analysis of a clinic register of 440 non-perinatal deaths in this community over 25 years (1984-2008) showed a mean age at death of 48 years with 25% of all deaths attributed to CVD. For deaths occurring between the ages of 15 to 54 (N = 216), 33% were attributed to CVD (unpublished data). The study setting is also typical of many primary health care (PHC) services in remote Aboriginal communities in Australia. The service: (i) is understaffed relative to identified needs [6], (ii) has a high turnover of non-Aboriginal health staff and (iii) has a predominant focus on acute care [7,8].

Between March and September 2005, the PHC team consisting of a GP, remote area nurses (RANs) and Aboriginal health workers (AHWs) conducted an outreach program of AHCs at 16 homelands, township residences, workplaces and public spaces (outside the community store and community council buildings). Prior to 2005, no AHCs had previously been undertaken and holistic CVD risk assessment was not part of routine PHC. Participation in the AHC program was on a voluntary basis. Information sheets were supplied and written informed consent was obtained from participants. In our study, the AHC constitutes a complex health service intervention comprising: (i) identification of patients with elevated CVD risk, (ii) chronic disease care planning - a patient centred consultation where patient education and brief interventions are delivered and treatment goals are negotiated with patients and (iii) follow-up of patients for chronic disease monitoring and further care planning.

During the AHC, CVD risk was assessed using the New Zealand Guidelines Group (NZGG) handheld chart based on review of the participant's medical records, AHC findings, adjustments for isolated extreme risk factors and ethnicity (increased by one risk category - 5%) [9]. Elevated CVD risk was defined as a NZGG chart risk of ≥10-15% chance of a CVD event over the next five years - the threshold where pharmacotherapy may be indicated (number needed to treat for 5 years to prevent one CVD event = 27) [9,10]. AHC participants with chronic disease diagnoses were offered follow-up care and care-planning through the PHC service consisting of a multidisciplinary team of GPs (N = 2.0), RANs (N = 10), AHWs (N = 3).

### Clinical Measures

#### Standardised clinical review

The same questionnaire, equipment and investigations for CVD risk assessment were used during the AHC and at a review date, on average, just over a year following participation in the AHC. Via an interviewer-administered questionnaire, we collected self-reported data on smoking status by asking: "Do you smoke tobacco?" (yes/no) [11]. Quantification of cigarettes smoked per day was undertaken using a four-point visual scale of increasing increments (5,10,15, 20 or more cigarettes per day) of the most commonly consumed tobacco product. Participants' weight was recorded on digital scales to the nearest 100 g, height to the nearest centimetre using a mounted stadiometer, and waist measured to the nearest millimetre with an inelastic tape using standard techniques [12]. Participants wore light clothing and had bare feet. Body Mass Index (BMI) was derived by dividing weight in kilograms by the square of the person's height in metres. Three blood pressure readings at one minute intervals were obtained on seated participants with an automated sphygmomanometer (Welch-Allyn: Spot Vital Signs 420TB-E1), using the correct cuff size for the upper arm circumference. The average of the second and third readings was calculated and hypertension defined as systolic blood pressure ≥ 140 mmHg and/or diastolic blood pressure ≥ 90 mmHg. Non-fasting blood samples were obtained. High density lipoprotein (HDL), total to HDL cholesterol ratio (lipid ratio) (colorimetric method) and blood glucose (hexokinase method) were measured on a Roche Cobas Integra 800 analyser. Type two diabetes was assessed by review of patient medical records or an indicative blood glucose level, confirmed by a subsequent oral glucose tolerance test [13].

#### Estimation of absolute CVD risk

While clinical decision making is based upon the use of handheld charts to identify patients with elevated CVD risk [9,14], these charts do not provide a precise estimation of CVD risk. To determine if there were any changes in the cohort's mean absolute CVD risk following the AHC, estimation of absolute CVD risk was undertaken for participants who completed both standardised clinical assessments (N = 58). We used the Framingham equations for coronary heart disease risk over five and ten years [15]. Although these equations have been demonstrated to underestimate absolute risk in one remote Aboriginal population [16], the purpose of this calculation was to identify 'relative' changes in the cohort's CVD risk subsequent to participation in the AHC (the intervention). A further issue with the equations is that they can only be used from the age of 30 [15]. Of the 58 participants completing both standardised clinical assessments, 11/58 (19%) were aged less than 30 years on the day of the AHC. For the purposes of absolute CVD risk calculation only, their ages were adjusted upwards so that they were assessed as if they were aged 30 on the day of the AHC. The same adjustment was applied to their age for the calculation of absolute risk when they completed the post-AHC review. To assess relative changes in the cohort's mean absolute CVD risk, three calculations were performed: (i) on the day of AHC participation (baseline risk), (ii) on the day of post-AHC review (on average, 1.2 years after the AHC), assuming no change in clinical parameters apart from age (expected post-AHC CVD risk) and (iii) on the day of post-AHC review using new clinical findings from the repeated standardised assessment (observed post-AHC CVD risk). A paired two-tailed t-test was then used to determine if the difference between the expected and observed measures of the cohort's mean absolute CVD risk at follow-up was statistically significant.

### Medical record auditing procedure

Inclusion criteria for the interrupted time-series (ITS) study were: (i) residence in the community for three years prior to the date of participation in the AHC and for three years following AHC participation (6 years in total), (ii) elevated CVD risk, (iii) participation in the AHC program (the intervention) and (iv) consent to the study.

Participants' medical records were audited for recorded delivery of NT Preventable Chronic Disease Strategy (PCDS) scheduled services for ischaemic heart disease and hyperlipidaemia (Table 1) [17], CVD related medication prescription, medication dispensing, PHC consultations, CVD events (myocardial infarction, stroke/transient ischaemic attack, coronary artery angioplasty/stenting) and iatrogenic events (medications errors, drug side effects, patient complaints).

**Table 1:** NT PCDS recommended clinical service items for ischaemic heart disease and hyperlipidaemia

**CVD secondary prevention services scheduled to be delivered once every 6 months**	
Consultations	With RAN or AHW, MO
Brief Interventions	Smoking, Nutrition, Alcohol, Physical activity, Emotional wellbeing
Clinical/lab services	Weight, Waist, Blood pressure, Lipids measurement
CVD secondary prevention services scheduled to be delivered once every 12 months	
Consultation	With Physician
Interventions	Influenza vaccination, Care planning
Clinical/lab services	ECG, ACR, FBE, LFT, EUC, BGL

Notes: NT PCDS = Northern Territory Preventable Chronic Disease Strategy, CVD = cardiovascular disease, RAN = remote area nurse, AHW = Aboriginal health worker, MO = medical officer, ECG = electrocardiograph, ACR = urinary albumin creatinine ratio, FBE = full blood examination, LFT = liver function tests, EUC = electrolytes, urea and creatinine, BGL = blood glucose level.

These outcomes were audited at six-monthly intervals for three years prior to the date of participation in the AHC and for three years following the date of participation in the AHC. Thus, a total of 12 six month intervals covering six years of PHC for each participant were audited. Within each six-month interval, each scheduled NT PCDS CVD preventive service (N = 20) was coded as delivered (1) or not (0). Services scheduled annually, if delivered, were coded as delivered both in the interval in which they occurred and the following six month interval. The proportion of the 20 NT PCDS scheduled CVD secondary prevention services delivered in each time interval was generated by summing services and dividing by 20. A sub-measure of six services with the strongest evidence for effectiveness (counselling for smoking cessation, measurement of blood pressure, lipids, blood glucose, annual influenza vaccination [18], and chronic disease care planning [19]) was also generated by summing these services and dividing by 6. A paper-based audit tool and audit protocol were designed by an expert panel and pre-piloted on a random sample of eight charts [20]. Participants' charts were audited by a senior GP (CPB) and a random sample of twenty charts (31%) was re-audited by CPB to evaluate intra-rater reliability. Reliability items (N = 12) audited comprised a range of (i) administrative data (N = 2) (AHC participation date, date of birth), (ii) categorical variables (N = 6) (recorded delivery of smoking brief intervention, blood pressure measurement, blood lipids, GP consultation, care planning, aspirin prescription), and (iii) numerical variables (N = 4) (systolic blood pressure, lipid ratio, number of GP consultations, proportion of prescribed medications dispensed).

### Statistical methods

Double data entry was used to minimise errors during data input. Means and proportions for CVD risk factors at the time of AHC and Pearson's chi square and two tailed independent samples t-tests for difference based on gender were performed. For participants who completed the post-AHC standardised clinical review, changes in CVD risk factors were tested using a paired two tailed t-test or McNemar's test. For the medical record audit variables, Cohen's Kappa statistic for dichotomous outcomes and quadratic weighted kappa statistic for ordinal outcomes were calculated to evaluate intra-rater reliability. Our criteria for satisfactory intra-rater reliability was a Kappa statistic of > 0.80 [21]. We calculated that a sample size of 60 participants would demonstrate a difference of 1.5 service items to be statistically significant with 80% power, based on a 2-sample paired t-test with alpha = 0.005 (to allow for multiple testing). Multiple paired two-tailed t-tests and repeated measures analysis (ANOVA) were performed to evaluate changes in CVD care outcomes over time. For the paired two-tailed t-tests used in the ITS, we used the Holm-Bonferroni adjustment to control for family wise error among the multiple t-tests to set an overall 0.05 family wise error rate [22]. All statistical analysis was undertaken using Stata software (version 9.2).

### Ethics approval

Ethics approval for this study was obtained from Charles Darwin University (H04053) and the NT Department of Health and Community Services (04/35). Ethics approval included independent approval by an Aboriginal ethics sub-committee and letters of support from the community controlled health board and outstation resource centre.

## Results

AHC participants were 301 adults (59% men, N = 177) aged 15 to 54 years, representing 23.4% of the eligible population (N = 1284) [23]. The sample age structure was similar to the most recent census profile (X^2 ^= 9.63, *P *= 0.2) [23]. During the AHC program, 75/301 (25%) participants were identified as having elevated CVD risk, with 63/75 (84%) having no previous documentation of elevated CVD risk. However, 31/63 (49%) of those 'newly detected' had sufficient pre-existing clinical and laboratory findings documented for elevated CVD risk to be identified prior to participation in the AHC. Women were significantly more likely to have elevated CVD risk overlooked prior to the AHC (X^2 ^= 6.5, *P *= 0.01).

### Interrupted time series findings

**Figure 1:**
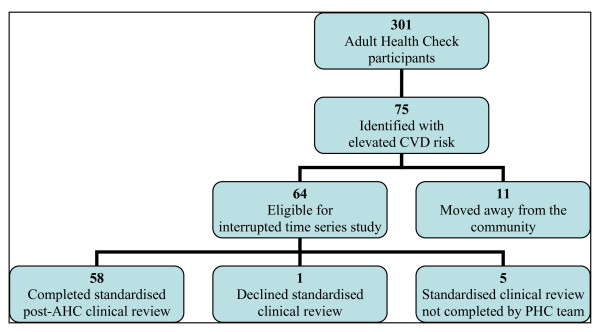
**Flowchart of study participants**. Notes: CVD = cardiovascular disease, AHC = adult health check, PHC = primary health care.

Eleven participants with elevated CVD risk were excluded from the ITS study because they had moved away from the community (Figure 1). The remaining 64 participants with elevated CVD risk were included. Among this cohort there was one death 18 months prior to the end of our study. The time series cohort provided 384 person-years of observation. Among participants, there were no significant differences between men and women in terms of their CVD risk factor profile (Table 2). Following AHC participation, 61/64 (95%) of the cohort underwent chronic disease care-planning with a medical officer between 1 and 555 days (mean 177 days) after the AHC. Two participants (3%) had a pre-existing care plan (that included a CVD related diagnosis) on the day of participation in the AHC. One participant did not undergo care planning during the study period.

**Table 2:** Time series cohort characteristics at the time of AHC participation (N = 64)

	Males	Females	All	P*
Participants N (%)	43 (67%)	21 (33%)	64	-
Mean age in years (SD)	39.6 (8.1)	42.5 (9.1)	40.6 (8.5)	0.21
Current smoker	81%	91%	84%	0.29
Type two diabetes	24%	43%	30%	0.12
Blood pressure ≥ 140/90 mmHg	21%	19%	20%	0.57
Total cholesterol ≥ 4.0 mmol/L	88%	86%	88%	0.53
HDL cholesterol ≤ 1.0 mmol/L	56%	62%	58%	0.43
Lipid ratio ≥ 5.0 (levels of total to HDL cholesterol)	56%	62%	58%	0.43
Mean NZGG 5-year CVD risk category† (SD)	4.5 (0.9)	4.6 (1.0)	4.5 (0.9)	0.66

Notes: AHC = adult health check, N = number, SD = standard deviation, HDL = high density lipoprotein, NZGG = New Zealand Guidelines Group, CVD = cardiovascular disease.

*Test for difference between men and women. Means were tested using a two-tailed independent samples t-test. Proportions were tested using Pearson's chi square test.

† NZGG risk categories: 4 = 10 to 15% over 5 years, 5 = 15 to 20% over 5 years, 6 = 20 to 25% over 5 years, etc.

‡ Calculated using the Framingham equations [15]. The equations have been shown to underestimate risk in one other remote Aboriginal population [16].

Repeat audits were completed between 43 and 79 days after the initial audit (mean 71 days). Kappa statistics for all twelve items covered by the repeat audit showed a high level of agreement (κ range: 0.88 to 1.0, observed versus expected agreement *P *< 0.001). Detailed results are available from the corresponding author.

**Figure 2:**
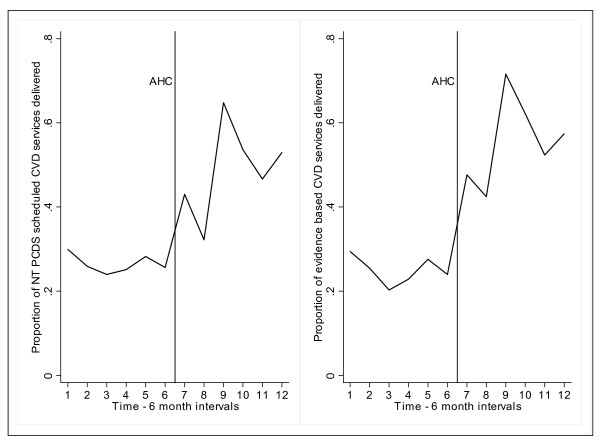
**Proportion of scheduled CVD secondary prevention services delivered over time**. Notes: CVD = cardiovascular disease, AHC = adult health check, NTPCDS = Northern Territory Preventable Chronic Disease Strategy. NT PCDS scheduled services comprise all 20 services detailed in Box 1 of this article. Evidence based CVD preventive services (N = 6): smoking advice, measurement of blood pressure, blood glucose, lipids, influenza vaccination and chronic disease care planning. Means are calculated for all participants in the time series study (N = 64). The vertical line mid-graph represents the intervention point: AHC participation.

Prior to the intervention, there were no significant changes between the first and subsequent time intervals in the recorded delivery of preventive care services for CVD (Figure 2 & Table 3). After the intervention and following Holm-Bonferroni adjustment, recorded delivery of scheduled CVD preventive care services increased significantly in all but one of the 6 month periods (repeated measures analysis *P *< 0.001). Recorded delivery of the subset of services with the strongest evidence base demonstrated a similar pattern to the total measure of NT PCDS CVD related preventive services. However, for evidence-based preventive care services for CVD, the improvement in recorded delivery was statistically significant in all time intervals following the intervention (table 4).

**Table 3:** Multiple paired t-tests of scheduled CVD services delivered over time compared to baseline (N = 64)

Time period 6 month intervals	Mean proportion of CVD services delivered	Difference from baseline	t-test	P*
Baseline	0.30	-	-	-
2	0.26	-0.04	-0.95	0.35
3	0.24	-0.06	-1.57	0.12
4	0.25	-0.05	-1.21	0.23
5	0.28	-0.02	-0.40	0.68
6	0.26	-0.04	-1.04	0.30
AHC participation (intervention)				
7	0.43	0.13	3.49	0.001
8	0.32	0.02	0.52	0.61
9	0.65	0.35	7.84	< 0.001
10	0.54	0.24	5.42	< 0.001
11	0.47	0.17	3.64	< 0.001
12	0.53	0.23	4.84	< 0.001

Notes: CVD = cardiovascular disease, AHC = adult health check, N = number.

*Two-tailed paired t-test for change in mean proportion of CVD services delivered compared with baseline.

**Table 4:** Multiple paired t-tests of 'evidence based' CVD services delivered over time compared to baseline (N = 64)

Time period 6 month intervals	Mean proportion of CVD services delivered	Difference from baseline	t-test	P*
Baseline	0.29	-	-	-
2	0.26	-0.04	-0.81	0.42
3	0.20	-0.09	-2.14	0.03
4	0.23	-0.06	-1.41	0.16
5	0.28	-0.01	-0.37	0.71
6	0.24	-0.05	-1.12	0.26
AHC participation (intervention)				
7	0.48	0.19	4.04	< 0.001
8	0.42	0.13	2.55	0.01
9	0.72	0.43	8.48	< 0.001
10	0.62	0.33	6.42	< 0.001
11	0.52	0.23	4.25	< 0.001
12	0.57	0.28	5.18	< 0.001

Notes: CVD = cardiovascular disease, AHC = adult health check, N = number.

*Two-tailed paired t-test for change in mean proportion of CVD services delivered compared with baseline.

**Figure 3:**
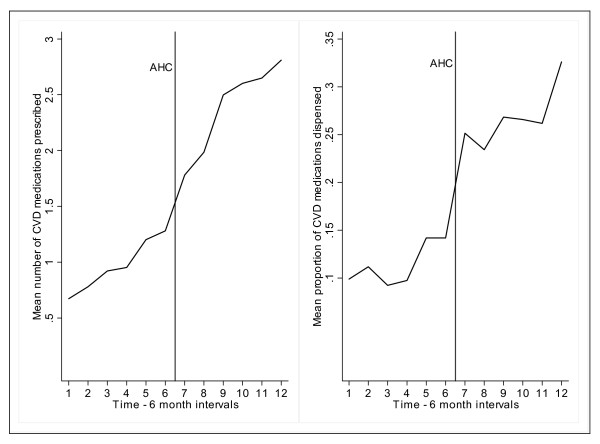
**CVD medication prescribing and recorded dispensing over time**. Notes: AHC = adult health check, CVD = cardiovascular disease. Means are calculated for all participants in the time series study (N = 64). The vertical line mid-graph represents the intervention point: AHC participation.

Prior to the intervention, there was an increase in CVD medication prescription and dispensing corresponding with incident CVD events among the cohort members (Figure 3). However, following Holm-Bonferroni adjustment, the changes between the first and subsequent time intervals prior to the intervention were not statistically significant. After the intervention, both the mean number and proportion of CVD related medication dispensed increased significantly (repeated measures analysis *P *< 0.001). At the start (3 years prior to participation in the AHC) of the time series 18/64 (28%) of participants were prescribed CVD related medication, rising to 56/63 (89%) by study end (three years after participation in the AHC). Anti-platelet drugs, lipid lowering agents, anti-hypertensives and oral hypoglycaemic agents were more likely to be prescribed at study end compared to the start of the study (Table 5). The mean proportion of prescribed CVD related medication dispensed at study end was 33%.

**Table 5:** Medications prescribed at study start (N = 64) and study end (N = 63)^†^

Drug class	Baseline N (%)	Study end N (%)	P*
Anti-platelet	3 (4.7%)	43 (68.3%)	< 0.001
Lipid lowering	4 (6.3%)	41 (65.1%)	< 0.001
ACEi/ARB	16 (25%)	40 (63.5%)	< 0.001
Oral hypoglycaemic	11 (17.2%)	21 (33.3%)	0.04
Beta blocker	3 (4.7%)	8 (12.7%)	0.09
Nitrate	2 (3.1%)	3 (4.8%)	0.49
Thiazide diuretic	0	2 (3.2%)	0.24
Calcium channel blocker	1 (1.6%)	1 (1.6%)	0.75

Notes: ACEi = angiotensin converting enzyme inhibitor, ARB = angiotensin receptor blocker.

* Chi Square test for change in proportion of participants prescribed each class of medication.

† There was one death in the period following the AHC.

Baseline = 3 years prior to date of AHC participation.

Study end = 3 years following date of AHC participation.

There were 2921 documented PHC consultations with medical officers, nurses, Aboriginal health workers and the visiting physician during the study period. Multiple two-tailed t-test analysis and repeated measures analysis did not demonstrate any significant changes in the mean number of PHC consultations between the first and subsequent time intervals.

There were 8 CVD events prior and 9 (including one death) following the intervention amongst the time series cohort. Six iatrogenic events were documented over the study period, 2 prior and 4 following the intervention. These included 2 prescribing errors by locum medical officers, a high international normalised ratio level in one patient prescribed warfarin, one minor vaccination reaction and 2 errors in pre-packaged medications. There were no significant trends in mean numbers of CVD or iatrogenic events between the initial and subsequent time intervals.

### Cohort CVD risk profile findings

Of the 64 participants in the time-series study (Figure 1), 58 (91%) completed a standardised post-AHC CVD review between 0.9 and 2.5 years (mean 1.2 years) following the AHC (Table 6). One participant declined the standardised review when offered. A further five participants, whilst having contact(s) with the PHC service, did not receive the standardised review. The six ITS participants who did not complete the repeat standardised clinical review were excluded from the cohort CVD risk profile analysis. Two participants had data missing for waist circumference or calculation of body mass index. At the post-AHC review, 6 participants self-reported that they had ceased smoking and of those who were smoking on both assessment dates, there was a statistically significant reduction in the self-reported number of cigarettes smoked per day. There were significant changes in terms of reduced waist circumference, increased HDL and decreased lipid ratio. There was no change in mean BMI or the prevalence of diabetes. At the post-AHC review, the mean estimated absolute CVD risk over ten years was 20% lower than expected (*P *= 0.004), compared with the mean estimated absolute CVD risk calculated on the presumption of no change in clinical parameters apart from age between the AHC and post-AHC review.

**Table 6:** CVD risk profile for study participants completing the post-AHC clinical review (N = 58), (figures are mean [SE] unless otherwise specified)

CVD risk factor	AHC	Review	P*
Current smoker N (%)	48 (83%)	45 (78%)	0.51
Quit smoking N (%)	-	6 (13%)	
Started or recommenced smoking N (%)	-	3 (30%)	
Category: cigarettes smoked per day† (N = 41)	3.5 (0.1)	2.6 (0.2)	< 0.001
Body mass index kg/m^2 ^(N = 56)	27.3 (0.9)	27.3 (0.8)	0.81
Waist circumference cm (N = 56)	98.3 (1.8)	96.4 (1.8)	0.04
Systolic blood pressure mmHg	128 (2.6)	124 (3.0)	0.2
Total cholesterol mmol/L	5.5 (0.2)	5.3 (0.2)	0.07
HDL cholesterol mmol/L	1.01 (0.03)	1.11 (0.04)	0.001
Ratio of total to HDL cholesterol	5.7 (0.2)	5.0 (0.2)	< 0.001
Type two diabetes N (%)	17 (29%)	17 (29%)	1.0
NZGG 5-year CVD risk category‡	4.5 (0.1)	4.3 (0.2)	0.11
Expected post-AHC absolute 5-year CVD risk %	4.3 (0.4)	4.6 (0.4)	< 0.001
Observed post-AHC absolute 5-year CVD risk %		3.6 (0.4)	
Difference in post-AHC absolute 5-year CVD risk %		1.0 (0.4)	0.007§
Expected post-AHC absolute 10-year CVD risk %	9.5 (0.8)	10.2 (0.8)	< 0.001
Observed post-AHC absolute 10-year CVD risk %		8.2 (0.7)	
Difference in post-AHC absolute 10-year CVD risk %		2.0 (0.7)	0.004§

Notes: CVD = cardiovascular disease, AHC = adult health check, SE = standard error, N = number, HDL = high density lipoprotein, NZGG = New Zealand Guidelines Group.

* Test for difference between findings at the AHC and at review. Means were tested by a paired two-tailed t-test. Proportions were tested using McNemar's test.

† Cigarettes per day (cpd) categories: 1 = 5cpd, 2 = 10cpd, 3 = 15 cpd, 4 = 20+ cpd.

‡ NZGG risk categories: 4 = 10 to 15% over 5 years, 5 = 15 to 20% over 5 years, 6 = 20 to 25% over 5 years, etc.

§ Two-tailed t-test for difference between observed absolute CVD risk at post-AHC review and expected (change in age only) absolute CVD risk at post-AHC review.

## Discussion

AHC-based holistic CVD risk assessment resulted in better identification of elevated CVD risk, improved delivery of preventive care services for CVD, intensification of pharmacotherapy for CVD and improvement in some intermediate CVD related clinical outcomes in this remote Aboriginal community.

Consistent with a recent study, we found significant gaps in screening, recognition of elevated CVD risk and initiation of risk reduction interventions prior to the AHC [24]. A strength of the AHC as an intervention, is the opportunity this presents PHC professionals to close this evidence-practice gap through the assessment and management of CVD risk [4]. The high proportion of those newly identified with elevated CVD risk, particularly those who had this overlooked during routine care, confirms the merits of holistic appraisal of CVD risk, rather than focussing on isolated risk factors [14]. A policy implication of our findings is the need to strengthen implementation of CVD risk assessment for Indigenous Australians [24]. Reliance upon the AHC as the only vehicle for CVD risk assessment may be inadequate because of the low uptake of the item [25].

Following identification of elevated CVD risk, recorded delivery of the NT PCDS scheduled preventive care services for CVD improved but also demonstrated some fluctuation. Reasons for this may include (i) the high turnover of nursing and medical staff [8], (ii) competing demands of the acute care workload [8], (iii) the time delay between the intervention and care-planning with a medical officer, and/or (iv) the concentration of preventive services within annual care planning consultations. Recorded delivery of preventive care services for CVD also improved without any increase in the mean number of PHC consultations. This finding attests to the potential of the AHC, based on a patient's documented risk assessment, to facilitate a shift from episodic acute care to an increased emphasis on prevention within a multi-disciplinary PHC team [4]. However, improvements in preventive care for CVD in remote communities are likely to require more PHC consultations, focused specifically on chronic illness care [19,26]. Research is required about how best to re-orient health centre systems and how to structure, recruit, train and retain the requisite workforce for chronic illness care in remote Aboriginal communities [8].

The high proportion (98%) of study participants who underwent care planning and the high proportion (89%) for whom pharmacotherapy was prescribed by study end indicates a high level of engagement with preventive care for CVD by this cohort. However, the increase in mean documented dispensing of CVD related medications over time, while also indicative of greater engagement with prevention, may have occurred for reasons other than the AHC. The gradual introduction of pre-packaged medications (multi-dose sachets and blister packs) over the study period may have contributed to increased medication dispensing. Our findings contrast with at least one previous study, where a primary health care system type intervention was not associated with intensification of medical treatment [27]. Nevertheless, the low mean proportion of medications dispensed (at best, 33% at study end), suggests that intervention with pharmacotherapy was suboptimal. This may explain, in part, the limited reduction in the cohort's CVD risk profile (for example blood pressure) despite the significant increase in medication prescription.

Compared with the only other published study on the AHC [28], our work contributes new knowledge in several important areas. First, our findings document the population burden of elevated CVD risk. This was not reported in the former study [28]. Second, we present prospective data on changes in participants' CVD risk profile suggestive of improved outcomes following AHC participation. Third, we demonstrate the high level of engagement of Aboriginal participants with preventive care through chronic disease care planning and assenting to pharmacotherapy. Fourth, we have identified significant PHC capacity constraints, in this research setting, to deliver optimal preventive care services for CVD through guideline scheduled clinical services and pharmacotherapy.

This last point is consistent with international experience - that the addition of new features, such as the AHC, to an unchanged PHC system focussed on acute care will have sub-optimal impacts on patients' chronic disease outcomes [19]. Transformational rather than incremental change may be required in remote Aboriginal communities to shift to an evidence-based chronic care model [26]. Interventions targeting health providers (such as the AHC) may need to be accompanied by systematic efforts to increase patients' knowledge, skills and confidence to manage their own conditions [29,30]. In the challenging setting of remote Aboriginal communities, specific strategies may be required to: (i) involve family members in care [19,31], (ii) train and retain a skilled medical and nursing workforce to provide high quality chronic illness care [8,19,26] and (iii) improve Aboriginal participation in the delivery of chronic disease care [8,31,32]. These remain the ongoing challenges for PHC practitioners, policy makers, health administrators and health researchers.

The relative reduction in the cohort's estimated absolute CVD risk attests to the potential of the AHC to contribute to reductions in the single largest cause of excess Aboriginal mortality [1,4], even within very challenging PHC settings. In addition, the reduction in absolute CVD risk may be underestimated in our study because of the significant reduction in the number of cigarettes smoked per day in this cohort [33]. The Framingham equations only include smoking status [14,15]. However, the improvement in participants' CVD risk profile must be interpreted with caution. In comparable settings, such improvements have been difficult to sustain in the long term [34].

A limitation of this study is that we were unable to report on clinical outcomes throughout the time series. Our clinical outcomes data, dependent on the PHC service's capacity to deliver preventive clinical services, did not meet accepted standards for reporting - 80% complete data at each time interval in a time series [21]. Furthermore, the unchanged mean number of CVD events prior and following the intervention suggest that the increase in preventive care and gains in intermediate CVD risk reduction were not sufficient to alter progression to end-stage disease in the short term. However, with a small sample size our study was not adequately powered to detect changes in CVD events. In addition, it is likely that the follow-up period for this study was too short to demonstrate such an effect.

Further limitations of this study include: 1) the involvement of the primary researcher/clinician (CPB) in the implementation of the intervention (the AHC) and the follow-up of clients identified with elevated CVD risk. The high level of engagement of this practitioner in the AHC, CVD assessment and ongoing care is likely to have been a significant factor in the observed increase in preventive care. An impact of this magnitude may not necessarily be expected with large scale implementation of the AHC. 2) The Framingham risk equations may underestimate 'absolute' CVD risk for Aboriginal Australians [16]. However, no studies have been published on Indigenous Australians applying the adjustments for ethnicity suggested by the New Zealand Guidelines Group [9,14]. While further work is required to develop accurate CVD risk assessment for Indigenous Australians, peak bodies advocate using the adjustments we have in this study [14]. 3) Potential sources of bias include the reliance on documentation of preventive care services for CVD delivered by PHC professionals. We don't believe the intervention (AHC) affected recording of preventive services because (i) the AHC is a discrete medical service for which documentation is independent of other chronic disease care documents, (ii) the source and methods of data collection were the same before and after the intervention and (iii) outcomes audited were objective (service provided or not, medication prescribed/dispensed or not). 4) Complex health service interventions are difficult to evaluate. The absence of a parallel control group - people identified with elevated CVD risk for whom intervention was not offered, may be considered a further limitation. However, within a population with a high burden of premature CVD related mortality, withholding intervention would be unethical and concealment of an intervention group would be difficult within a single PHC service with multiple practitioners. The historical controls provided by an interrupted time series study provide a robust alternative and our study complies with eligibility criteria for inclusion in systematic reviews [21].

## Conclusions

We conclude that CVD risk assessment during the AHC can be an effective intervention towards better and earlier detection of elevated CVD risk and population reductions in CVD risk but significant challenges remain to improve preventive care for CVD. Further work is required to understand how to establish effective and sustainable chronic disease care in remote Aboriginal PHC settings to decrease excess CVD mortality.

## Competing interests

The authors declare that they have no competing interests.

## Authors' contributions

CPB conceived the study, undertook the data collection, statistical analysis and had primary responsibility for drafting the manuscript under the primary supervision of RSB. CMC, RDC, KOD and RAM assisted with the study design, presentation, interpretation of results and critically reviewed the manuscript. HLM and CG assisted the conduct and implementation of the project in the community setting, contributed a community interpretation of the results and reviewed the manuscript. AJE provided statistical advice, supervised the statistical analysis and critically appraised the manuscript. All authors read and approved the final manuscript.

## Pre-publication history

The pre-publication history for this paper can be accessed here:

http://www.biomedcentral.com/1472-6963/11/24/prepub
